# Visual Subfield Progression in Glaucoma Subtypes

**DOI:** 10.1155/2018/7864219

**Published:** 2018-03-21

**Authors:** Wei-Wen Su, Shian-Sen Hsieh, Shih-Tsung Cheng, Cheng-Wen Su, Wei-Chi Wu, Henry Shen-Lih Chen

**Affiliations:** ^1^Department of Ophthalmology, Chang Gung Memorial Hospital, Taoyuan, Taiwan; ^2^Department of Internal Medicine, Chang Gung Memorial Hospital, Taoyuan, Taiwan; ^3^Department of Cardiology, Buddhist Tzu Chi General Hospital Taipei Branch, Xindian, New Taipei City, Taiwan

## Abstract

**Purpose:**

To investigate visual field progression pattern and factors associated with progression in patients with primary open-angle glaucoma (POAG), normal-tension glaucoma (NTG), and chronic angle-closure glaucoma (CACG).

**Methods:**

The raw data of the 30-2 Humphrey Field Analyzer from glaucoma patients with definite visual field progression were processed with pointwise linear regression (PLR) analysis. The rate of change of retinal threshold sensitivity in the ten glaucoma hemifield test (GHT) zones, the upper and the lower hemifields, and the whole field was evaluated and was correlated with patients' basic demographic data.

**Results:**

An average follow-up of 6.94 ± 2.69 years that showed the rate of change of visual field threshold sensitivity was correlated with the peak posttreatment intraocular pressure (IOP) and the long-term IOP fluctuations in all GHT zones except in the inferior arcuate area. The baseline IOP, the trough posttreatment IOP, the refractive status, and the CCT were not correlated with VF progression.

**Conclusion:**

The rate of visual field progression was correlated with the peak posttreatment IOP and the long-term IOP fluctuation but with subfield differences.

## 1. Introduction

Glaucoma, one of the leading causes of blindness worldwide, is a group of ocular diseases characterized by a specific pattern of abnormalities of the optic nerve head and the retinal nerve fiber layer, with corresponding visual field loss. The key feature of glaucoma pathogenesis is progressive degeneration of retinal ganglion cells that leads to irreversible optic nerve damage and ultimately vision loss. Accurate and timely detection and quantification of disease progression are crucial to ensure appropriate clinical management to preserve long-term vision.

The Humphrey Field Analyzer (HFA; Carl Zeiss Meditec, Dublin, CA, USA) is a commonly used measurement to detect the extent of functional loss and disease worsening in patients with glaucoma. Several methods have been applied to identify visual field deterioration [1–4], categorized as either event-based or trend-based analyses. The event-based analysis compares the differences between baseline and follow-up examinations and matches to the test-retest variability of the reference sample of patients. Progression is flagged when the observed changes exceeds the predefined test-retest limits [5]. The trend-based analysis detects progression as changes over time in serial measurements and provides useful information in the long term, since glaucoma is a progressive disease. The mean deviation (MD) slope and the visual field index (VFI) slope of the Humphrey Field Analyzer are typical global indexes for determining and predicting glaucoma progression; both are very helpful in clinical practice [6]. However, early glaucoma changes are usually trivial and localized, which cannot be detected efficiently by the global indexes. Pointwise linear regression (PLR) that reveals the regression of retinal threshold sensitivity versus time at each test location is another way to evaluate visual field progression. A test location is considered to be progressing if it fulfills specified slope and significance criteria. Visual field progression is defined according to the number of test locations showing the given magnitude of slope and statistical significance [5, 7, 8]. PLR has been used frequently in research settings to detect visual field progression [9–11].

Randomized clinical trials had identified intraocular pressure (IOP) as a major risk factor for the development and progression of glaucoma [12–17]. Refractive status and central corneal thickness (CCT) had also been reported to be predictive for the development or progression of glaucoma [18–22]. Different glaucoma subtypes may progress at different rates [23, 24], and there are subfield differences in response to common causative factors [25]. The primary objective of this report is to evaluate the visual subfield progression rate in glaucoma subtypes and to identify clinical factors associated with their progression.

## 2. Materials and Methods

### 2.1. Participants

**Figure 1:**
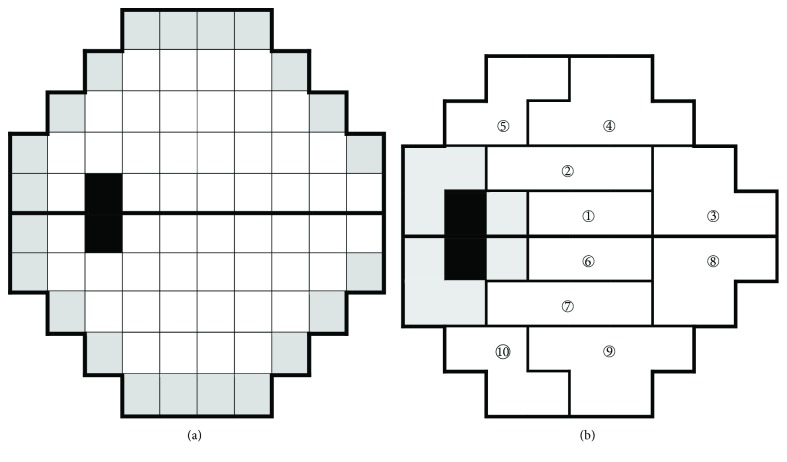
Location of (a) the 52 test points and (b) the 10 GHT zones of the 30-2 SITA program enrolled for pointwise linear regression analysis. Cells in gray and black are edge points and points corresponding to the blind spot that were removed from calculation. Digit in circle indicates the GHT zone number. GHT: glaucoma hemifield test.

The raw data of retinal sensitivity (in dB) from the Humphrey Field Analyzer 30-2 Swedish interactive threshold algorithm (SITA) program (Carl Zeiss Meditec, Dublin, CA, USA) between 2001 and 2014 were extracted for analysis. Visual fields with poor reliabilities, defined as fixation losses > 20% or false positive errors > 15% or false negative errors > 33%, were excluded from analysis. For reliable tests, those with five or more follow-ups were enrolled for progression analysis. After eliminating edge points (except the two nasal-most points across the horizontal midline) and the two points corresponding to the blind spot, the rate of change of retinal threshold sensitivities in each of the 52 test points (Figure 1(a)) was calculated with pointwise linear regression. Progressive fields were defined as having two or more significant progressive points in the same hemifield; each had a slope of sensitivity of less than −1.0 dB/year at *P* < 0.01, which was a frequently applied criterion for PLR analysis [26]. Eyes with definite visual field progression were recruited for further analysis. The rate of change of VF threshold sensitivity in each 52 test points, the 10 glaucoma hemifield test (GHT) zones (clusters of locations based on GHT) (Figure 1(b)), the superior and the inferior hemifield, and the whole field were investigated.

The recruited eyes should present typical glaucomatous disc changes and corresponding visual field losses. The interval between tests were generally every 6 months. Eyes with previous ocular trauma or ocular diseases other than glaucoma were excluded. The enrolled eyes were classified into 3 groups according to the angle structure and the baseline IOP. The primary open-angle glaucoma (POAG) group had open anterior chamber angle on gonioscopy, and baseline IOP exceeded 21 mmHg. The normal-tension glaucoma (NTG) group was identical to POAG except that the baseline IOP never exceeded 21 mmHg measured at varying intervals between 8 AM to 5 PM. The chronic angle-closure glaucoma (CACG) group had closed anterior chamber angles despite patent iridotomies. All glaucoma patients were under medical treatment.

The demographic information including the patients' age of onset, gender, baseline IOP, peak and trough posttreatment IOP during the entire follow-up period (maxIOP and minIOP), long-term IOP fluctuation (IOPf, the difference between the peak and trough IOP), central corneal thickness (CCT), refractive status, baseline and final MD, and pattern standard deviation (PSD) of the visual field tests was collected for correlation analyses. The study followed the tenets of the Declaration of Helsinki and was approved by the Institutional Review Board of Chang Gung Memorial Hospital.

### 2.2. Statistics

Data were expressed as mean ± standard deviation (SD) for continuous variables and percentage for categorical variables. The computational statistical environment R (http://www.r-project.org) was used to carry out large-scale pointwise linear regression analyses. For subtypes of glaucoma, continuous variables were compared among the POAG, the NTG, and the CACG group by one-way analysis of variance (ANOVA) followed by Bonferroni multiple comparison test. For relationship between parameters and visual field progression in each GHT zones and hemifields, the Pearson correlation coefficient was calculated. A *P* < 0.05 was considered statistically significant.

## 3. Results

**Figure 2:**
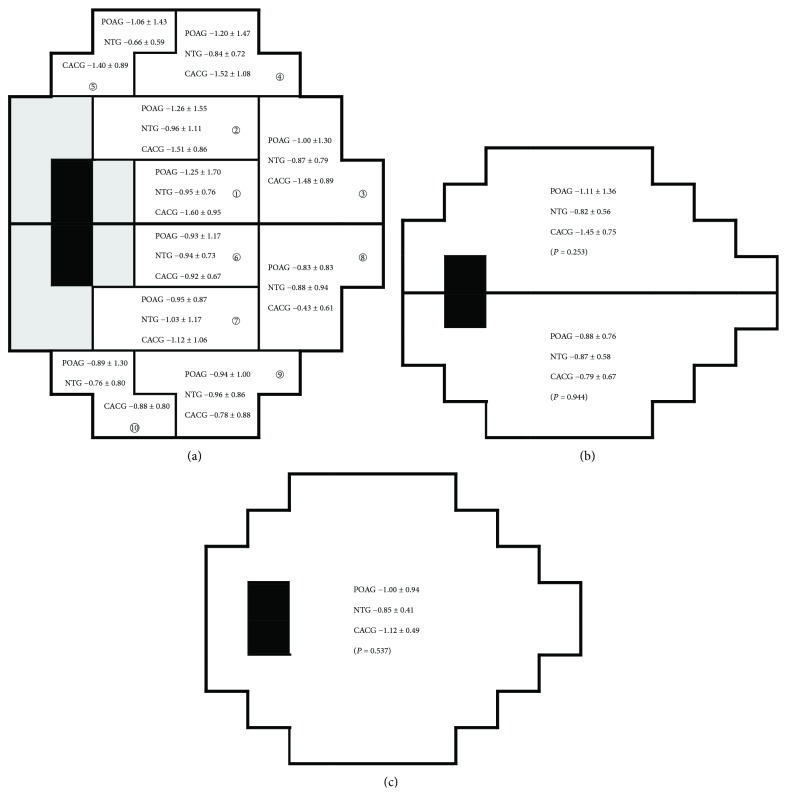
The annual regression slope of VF threshold sensitivity (dB/year) of (a) the 10 GHT zones, (b) the superior and the inferior hemifields, and (c) the whole field in the three glaucoma subtypes. Digit in circle indicates the GHT zone number.

Sixty-five eyes from 65 individuals were enrolled in the study, among which 29 were POAG, 27 were NTG, and 9 were CACG.  Table 1 lists the basic demographics of the three groups. Patients with POAG were younger, excessively myopic, and had thicker central corneal thickness (CCT). Patients with CACG were older, more hyperopic, and had thinner CCT. Patients with NTG were older at onset than patients with POAG and were less myopic (though statistically insignificant, *P* = 0.09). Greater IOP fluctuations were noted in the POAG and the CACG groups. The baseline MD of the visual field did not differ in the three groups.  Figure 2 shows the rate of change of VF threshold sensitivity in the 10 GHT zones, the superior and the inferior hemifields, and the whole field. An averaged follow-up of 6.94 ± 2.69 years showed a faster progression in the superior hemifield in patients with POAG and CACG. The NTG patients demonstrated equal but slightly faster progression in the inferior hemifields.

**Figure 3:**
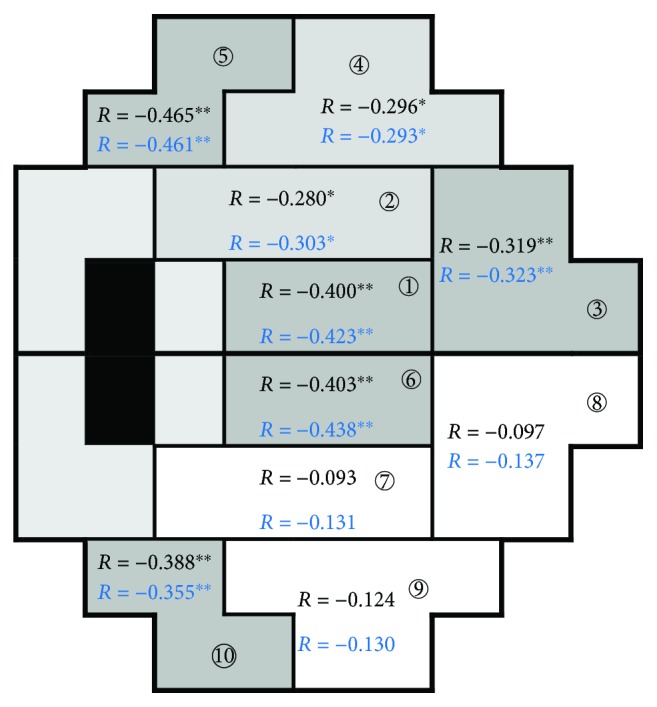
The correlation of the maximal posttreatment IOP and the long-term IOP fluctuations with VF progression in the 10 GHT zones. ^∗^*P* < 0.05; ^∗∗^*P* < 0.001. Digit in circle indicates the GHT zone number. Darker cells indicate a more significant correlation.

The age of onset, CCT, refractive status, baseline IOP, and minIOP were not associated with glaucoma progression. The rate of progression was associated with maxIOP and IOPf in all GHT zones except zone 7, zone 8, and zone 9 (Figure 3). The progression was not correlated with the baseline MD but mildly with the final MD.

## 4. Discussion

In the current study, the average annual progression rate was −1.00 ± 0.94 dB in POAG, −0.85 ± 0.41 dB in NTG, and −1.12 ± 0.49 dB in CACG. Compared to previous investigations, the OHTS reported rate of change in POAG eyes was −0.26 ± 0.36 dB/year [27], the EMGT reported between −0.36 to −1.31 dB/year [28], and the CNTGSG reported between −0.41 to −0.90 dB/year [29]. Studies from Japan showed the progression rates in treated NTG patients were between −0.1 and −0.35 dB/year [22, 30, 31]. The various results among different study groups suggested that high interpatient variability exists in visual field progression.

Myopia had been identified as a risk factor for the development and progression of POAG [21, 32, 33]. Visual field defects worsened with the increase of myopia in patients with POAG, and severe myopia can be a significant risk factor for subsequent visual field loss [34, 35]. On the other hand, some studies showed that the extent of myopia was a significant positive prognostic factor for glaucoma progression [22, 36]. Araie et al. reported that the less extent of myopia was a significant risk factor for visual field progression in patients with NTG [37]. In the current study, the degree of myopia was not correlated with VF progression in all the GHT subfields. The extent to which myopia affects the progression of OAG remains to be clarified.

CCT had also been identified as a risk factor for glaucoma. Correlation between thinner CCT and progressive visual field loss had been reported [15, 38], which might be independent of IOP [39]. In patients with ocular hypertension, the OHTS demonstrated that CCT is an important and independent risk factor for progression to initial glaucoma damage [14]. Visual field progression was significantly associated with thinner CCT both in patients with POAG and NTG [40, 41]. Conversely, Chauhan et al. reported that once glaucomatous damage was present, CCT was unlikely to be a useful predictive index for visual field and optic disc progression [42]. Jonas et al. reported that the amount of glaucomatous optic nerve damage correlated significantly with a thin central cornea, but progression of glaucomatous optic nerve neuropathy was independent of central corneal thickness [43]. Our report demonstrated that although the CCT differed among the three glaucoma subtypes, correlation between CCT and VF progression was not present. Since all our patient had definite visual field progression, CCT may not be a useful correlated factor in this group of patients.

The level of IOP has been shown to be the most important factor for both glaucoma development [14, 44, 45] and progression [15, 46, 47]. In the EMGT, the magnitude of initial IOP reduction was a major factor influencing outcome [46]. However, in the CNTGSG, the untreated level of intraocular pressure did not affect the rate of untreated disease progression, despite their known influence on prevalence [13]. The current study demonstrated that only the peak posttreatment IOP and the long-term IOP fluctuations were associated with glaucoma progression. Furthermore, subfield analysis showed that the progression in certain area of the visual field, such as the GHT zones 7, 8, 9 (the inferior arcuate area) was not correlated with any parameter of IOP. This finding suggested that the rate of change in certain visual subfield (the inferior arcuate area) may be less sensitive to IOP changes. Visual field progression in the inferior peripheral area was reported to be significantly correlated with peripheral vascular endothelial function, suggesting regional vulnerability to vascular dysregulation [48]. The current study further responds to this possibility.

## 5. Conclusion

In general, the superior fields progressed faster than the inferior fields. Among common risk factors for glaucoma, only the peak posttreatment IOP and the long-term IOP fluctuation were correlated with visual field progression. Progression in the inferior arcuate area was not correlated to any IOP parameters, suggesting regional difference in the visual subfield in terms of vulnerability to IOP changes.

## Figures and Tables

**Table 1:** Basic demographics between the three glaucoma subtypes.

	POAG (*n* = 29)	NTG (*n* = 27)	CACG (*n* = 9)	*P* value
Number of progressive points	8.45 ± 7.99	5.37 ± 3.14	6.67 ± 5.36	0.171
M : F	20 : 9	15 : 12	5 : 4	0.556
Age of onset	41.52 ± 12.69	50.16 ± 10.97	61.02 ± 5.41	<0.001
F/u years	7.28 ± 2.76	6.37 ± 2.87	7.53 ± 1.58	0.353
Baseline IOP (mmHg)	24.77 ± 5.80	14.54 ± 3.20	20.97 ± 6.95	<0.001
IOP min (mmHg)	12.02 ± 3.15	9.96 ± 1.85	9.62 ± 1.98	0.005
IOP max (mmHg)	26.05 ± 8.72	16.53 ± 2.57	22.28 ± 2.19	<0.001
IOP fluctuation (mmHg)	14.03 ± 9.23	6.57 ± 2.07	12.66 ± 1.59	<0.001
CCT (*μ*m)	554.56 ± 37.11	532.69 ± 37.12	520.00 ± 34.84	0.028
SE (D)	−6.73 ± 4.74	−4.26 ± 3.97	+0.06 ± 2.08	<0.001
MD baseline (dB)	−9.51 ± 7.28	−6.42 ± 6.34	−10.83 ± 7.44	0.140
MD final (dB)	−15.08 ± 9.01	−10.97 ± 7.12	−20.86 ± 7.25	0.007
